# Glass polymorphism in glycerol–water mixtures: II. Experimental studies

**DOI:** 10.1039/c5cp08069j

**Published:** 2016-04-05

**Authors:** Johannes Bachler, Violeta Fuentes-Landete, David A. Jahn, Jessina Wong, Nicolas Giovambattista, Thomas Loerting

**Affiliations:** a Institute of Physical Chemistry , University of Innsbruck , A-6020 Innsbruck , Austria . Email: thomas.loerting@uibk.ac.at; b Brooklyn College of the City University of New York , Brooklyn , NY 11210 , USA; c PhD Programs in Physics and Chemistry , the Graduate Center of the City University of New York , New York , NY 10016 , USA

## Abstract

We report a detailed experimental study of (i) pressure-induced transformations in glycerol–water mixtures at *T* = 77 K and *P* = 0–1.8 GPa, and (ii) heating-induced transformations of glycerol–water mixtures recovered at 1 atm and *T* = 77 K. Our samples are prepared by cooling the solutions at ambient pressure at various cooling rates (100 K s^–1^–10 K h^–1^) and for the whole range of glycerol mole fractions, *χ*_g_. Depending on concentration and cooling rates, cooling leads to samples containing amorphous ice (*χ*_g_ ≥ 0.20), ice (*χ*_g_ ≤ 0.32), and/or “distorted ice” (0 < *χ*_g_ ≤ 0.38). Upon compression, we find that (a) fully vitrified samples at *χ*_g_ ≥ 0.20 do not show glass polymorphism, in agreement with previous works; (b) samples containing ice show pressure-induced amorphization (PIA) leading to the formation of high-density amorphous ice (HDA). PIA of ice domains within the glycerol–water mixtures is shown to be possible only up to *χ*_g_ ≈ 0.32 (*T* = 77 K). This is rather surprising since it has been known that at *χ*_g_ < 0.38, cooling leads to phase-separated samples with ice and maximally freeze-concentrated solution of *χ*_g_ ≈ 0.38. Accordingly, in the range 0.32 < *χ*_g_ < 0.38, we suggest that the water domains freeze into an interfacial ice, *i.e.*, a highly-distorted form of layered ice, which is unable to transform to HDA upon compression. Upon heating samples recovered at 1 atm, we observe a rich phase behavior. Differential scanning calorimetry indicates that *only* at *χ*_g_ ≤ 0.15, the water domains within the sample exhibit polyamorphism, *i.e.*, the HDA-to-LDA (low-density amorphous ice) transformation. At 0.15 < *χ*_g_ ≤ 0.38, samples contain ice, interfacial ice, and/or HDA domains. All samples (*χ*_g_ ≤ 0.38) show: the crystallization of amorphous ice domains, followed by the glass transition of the vitrified glycerol–water domains and, finally, the melting of ice at high temperatures. Our work exemplifies the complex “phase” behavior of glassy binary mixtures due to phase-separation (ice formation) and polyamorphism, and the relevance of sample preparation, concentration as well as cooling rates. The presence of the distorted ice (called “interphase” by us) also explains the debated “drift anomaly” upon melting. These results are compatible with the high-pressure study by Suzuki and Mishima indicating disappearance of polyamorphism at *P* ≈ 0.03–0.05 GPa at *χ*_g_ ≈ 0.12–0.15 [*J. Chem. Phys.*, 2014, **141**, 094505].

## Abstract 2

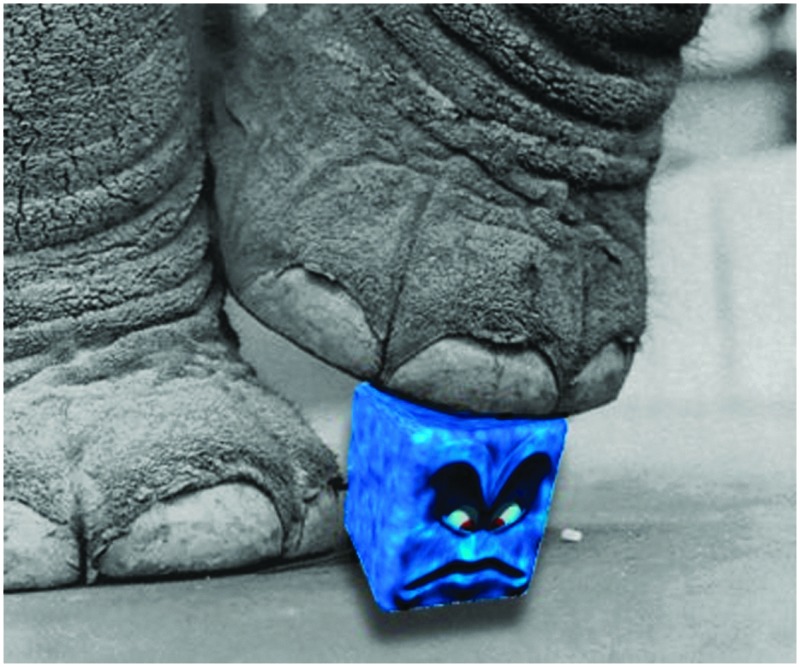
We here study pressure-induced amorphization and polyamorphic transitions in frozen bulk glycerol–water solutions experimentally.

## 1. Introduction

The occurrence of (at least) two distinct amorphous ices was discovered in 1984 by Mishima *et al.* ^1,2^ It has been proposed^3^ that these amorphous ices, low-density (LDA) and high-density amorphous (HDA) ice, represent the glassy counterparts of two distinct deeply supercooled liquids in the one-component system H_2_O. Also other glassy materials may occur in a range of different structural states that can be described using excess functions, such as excess volume, enthalpy or entropy. In other words, a range of different relaxation states can be accessed in any glassy material. The phenomenology associated with amorphous ices is, however, quite different from the phenomenology known in traditional glass physics. Most notably, latent heat is released^4^ and the volume suddenly increases by about 25%^2^ upon converting HDA to LDA. Furthermore, HDA and LDA can be reversibly converted into each other with hysteresis in compression and decompression experiments.^5,6^

The LDA–HDA transition is not progressing continuously, but discontinuously, *i.e.*, an interface between HDA and LDA develops,^6,7^ and X-ray^6^ and neutron diffraction patterns^8^ showing two distinct halo peaks can be recorded. The character of the HDA ↔ LDA transition is highly similar to the character of a first-order transition such as melting/freezing of ice. Whereas the latter represents the transition between two thermodynamically stable phases, the HDA ↔ LDA transition takes place in the thermodynamic stability zone of crystalline ices. However, the HDA → LDA transition takes place many orders of magnitude faster than crystallization both at 1 atm near 130 K^9^ and at 0.5 GPa near 80 K.^10^ In particular, the amorphous–amorphous transition takes place on the time scale of seconds or less, whereas no signs for the transformation to crystalline ices could be observed on the time scale of hours. This makes the case for “polyamorphism” and the possibility of more than one liquid in a one component system. Of course, slow relaxation effects take place in both amorphous ices in addition to the polyamorphic transition and the possibility of crystallization, so that the overall process is quite complex as also noted by Gromnitskaya *et al.* ^11^ Three fundamental processes, namely reversible and irreversible relaxation and crystallization, were carefully disentangled by Seidl *et al.* ^12,13^ They report the glass transition temperatures of HDA based on the study of its volumetric behavior as a function of pressure and show that the HDA matrix becomes an ultraviscous liquid just prior to the crystallization temperature. These results are confirmed from calorimetric^14,15^ as well as from dielectric measurements.^9,16,17^

However, near about 150–160 K crystallization rates grow rapidly so that the ultraviscous liquid can no longer be studied on the second time scale in bulk water.^18^ In order to avoid crystallization aqueous solutions are studied, among other methods. In this work we focus on glycerol–water mixtures and investigate polyamorphism, *i.e.*, glass polymorphism, from the experimental side. Complementary numerical simulations are reported in the companion article. From the experimental side, there has been quite some interest in vitrified glycerol–water solutions in the last few years.^19–27^ One of the debated issues concerns the transformation underwent by glycerol–water solutions near 170 K and 0.17 mole fraction. While Murata and Tanaka interpret their Raman data to indicate an isocompositional liquid–liquid transition,^24^ Suzuki and Mishima interpret their Raman data to indicate crystallization near 170 K and 0.17 mole fraction.^26^ However, Suzuki and Mishima find evidence for polyamorphism in solutions up to about 0.12–0.15 mole fraction of glycerol and claim a liquid–liquid transition ending in a critical point at ∼0.03–0.05 GPa and ∼150 K for these solutions.^26^ That is, above 0.12–0.15 mole fraction polyamorphism disappears. By contrast, Murata and Tanaka estimate the critical concentration to be at 0.032 mole fraction, below which the liquid–liquid transition disappears.^24^ At 0.17 mole fraction and 170 K the sample is in a quite different state according to the two studies: Suzuki and Mishima regard it as a fluctuating, supercritical one-component liquid on the verge of crystallization, while Murata and Tanaka see evidence for nucleation and growth of one liquid inside another liquid. The view of Suzuki and Mishima is backed by Popov *et al.* ^25^ Based on the study of glycerol–water solutions cooled at ambient pressure they divide the phenomenology in three ranges: (a) complete vitrification at 0.28–1.00 mole fraction, (b) cold-crystallization upon heating at 0.15–0.28 mole fraction and (c) crystallization of ice upon cooling at 0.00–0.15 mole fraction. According to Popov *et al.*, Murata and Tanaka have observed cold-crystallization rather than a liquid–liquid transition. We agree with the assessment by Popov *et al.* as well as Suzuki and Mishima.

In the present work we expand on the work by Popov *et al.* and study glycerol–water solutions after cooling to 77 K and pressurization to 1.8 GPa. In pure water, this procedure results in pressure-induced amorphization of hexagonal ice I_h_ and formation of HDA.^1^ Analysis of the sample after quench-recovery to 77 K and 1 atm using differential scanning calorimetry (DSC) reveals first the latent heat released on the polyamorphic transition HDA → LDA, which is then followed by the release of latent heat upon crystallization, LDA → ice I_c_. In parallel to the experimental study we conducted a numerical simulation of glycerol–water solutions cooled to 77 K and then pressurized. Also in the numerical simulation the resulting state is ultimately HDA. In both parts, we aim at the study of the influence of increasing mole fractions of glycerol on the stability of HDA and LDA, and hence a deeper understanding of polyamorphism. This also complements the study by Suzuki and Mishima, who were investigating polyamorphism in glycerol–water by studying the HDA–LDA transition under pressure. Our study, therefore, allows for a comparison of the HDA-like solution formed after pressure-induced amorphization from crystallized solution and the HDA-like solution formed upon compression of amorphous solution.

## 2. Experimental methods

We follow a protocol similar to the one employed in the accompanying computer simulation study.^28^ That is, we cool aqueous glycerol solutions at *P* = 1 atm to liquid nitrogen temperature (*T* = 77 K), followed by compression at 77 K to 1.8 GPa in a piston-cylinder setup. The setup, including the use of indium linings to avoid friction,^1^ is identical to the one employed in many of our previous studies; see, *e.g.*, ref. 6, 12, 15, 29–31. All pressurization experiments are performed using samples of 600 μl which are compressed uniaxially at 77 K within the bore of a steel cylinder. In the case of pure water, this protocol results in pressure-induced amorphization (PIA) of ice, leading to the formation of HDA.^1^

The samples obtained at high pressure are then decompressed to ambient pressure and taken out of the steel-cylinder, which remains immersed in liquid nitrogen in the whole procedure. *Ex situ* characterization of these samples is done by loading about 10–20 mg of the sample into an aluminum crucible, again, under liquid nitrogen. The crucible is finally cold-loaded into the DSC instrument (DSC 8000, Perkin Elmer) and heated at a rate of 30 K min^–1^ from 93 K to 300 K. The heating protocol is performed twice. In the first scan we measure the onset temperatures, latent heats, and heat capacity changes associated to the amorphous–amorphous, crystallization, and glass transitions. The second scan serves as a baseline and to disentangle reversible thermal effects from irreversible ones. Phases were identified by X-ray diffraction and phase transformations correlated with DSC exotherms and sudden volume changes in several previous studies.^1,2,32^ Thus, we here base our phase identification solely on DSC and volume changes.

### Nature of the samples at low-temperatures

Dilute solutions cooled at fast rates, 1 K s^–1^ < *q*_c_ < 1000 K s^–1^, phase-separate into ice domains and concentrated glycerol–water glassy domains (see, *e.g.*, 17, 20, 25 and 33–35) while, at high concentrations, cooling results in homogeneous glassy mixtures.^25^ In order to avoid crystallization in pure water and dilute solutions, ultrafast cooling experiments (“hyperquenching”) at *q*_c_ ∼ 10^6^–10^7^ K s^–1^ are required,^36–38^ which is beyond the scope of the present work. Here we consider two cooling rates, a fast rate of *q*_c_ ≈ 100 K s^–1^ and a slow rate of *q*_c_ ≈ 10–50 K h^–1^. The fast rate is achieved by pipetting 600 μl of solution into a steel cylinder cooled to *T* = 77 K. The slow cooling experiments are done by placing the solution vials in a Styrofoam box in a freezer (193 K) overnight.

We use the fast rate to prepare samples within the whole range of glycerol mole fractions, *χ*_g_ = 0.00–1.00. As we show in the next section, during pressurization at 77 K, samples with *χ*_g_ ≤ 0.20 exhibit PIA, at least partly. Hence, at these concentrations, samples cooled at *q*_c_ ≈ 100 K s^–1^ contain ice domains large enough to experience PIA. The remainder of the sample is composed of freeze-concentrated glycerol–water solutions, which do not crystallize, but vitrify. This is consistent with earlier work on vitrification/crystallization of glycerol–water solutions, which is based on diffraction, thermal analysis and broadband dielectric spectroscopy characterization of the cooled samples.^17,20,25,33–35^ The eutectic composition in glycerol–water mixtures is located at *χ*_g_ = 0.28^39^ (see also Fig. 7). Typically, glass formation ability is best near the eutectic composition. Indeed, our fast-cooled solutions vitrify homogeneously at *χ*_g_ > 0.20. However, for our slow-cooled solutions we see signs of PIA, and hence crystallization, even slightly above and below the eutectic composition, *i.e.*, at 0.20 ≤ *χ*_g_ ≤ 0.30 (see next section).

Because of supercooling, the freeze-concentrated solution can actually reach compositions of *χ*_g_ = 0.38–0.40, depending on the cooling rate employed.^19,34^ Freeze-concentration to *χ*_g_ > 0.38–0.40 is not possible because at these compositions the solution has the same glass-transition temperature *T*_g_ as the homogeneous glass (see Fig. 7 here and Fig. 4 in ref. 19). In other words, more ice cannot crystallize within these samples upon continued cooling because once the glassy–water mixture domains reach the concentration *χ*_g_ = 0.38–0.40, they become immobile below *T*_g_. This composition is known as the maximally-freeze concentrated solution (MFCS) or “critical concentration” (which we regard to be a misleading term in this context, unrelated to criticality). The glass transition temperature associated with the glycerol–water MFCS is *T*_g_ ≈ 175 K for rates of 20 K min^–1^ ^40^ and *T*_g_ ≈ 165 K for rates of 0.08 K min^–1^.^19^ As a result, all solutions of *χ*_g_ > 0.38–0.40 vitrify completely upon cooling, whereas for solutions of *χ*_g_ < 0.38–0.40, it is a matter of cooling rate whether they vitrify homogeneously or phase-separate. As mentioned above, in the case of our fast cooling rate, we observe homogeneous vitrification for all solutions of *χ*_g_ ≥ 0.20. The slow cooling rate used in our study is to allow for ice crystallization for solutions in the range 0.20 ≤ *χ*_g_ ≤ 0.30. In order to vitrify solutions at *χ*_g_ < 0.20 upon cooling it would be necessary to employ the hyperquenching technique at 1 atm or the technique of quenching the solutions at high-pressure, *e.g.*, 0.3 GPa. Both procedures would allow investigating the polyamorphic transition of the solvent mixed homogeneously with glycerol, *i.e.*, the LDA-to-HDA transition upon pressurization and the HDA-to-LDA transition upon depressurization (at elevated temperatures). The approach of quenching at high-pressure conditions, namely 0.3 GPa, was used previously by Suzuki and Mishima.^26^ By contrast to the latter work^26^ we here use bulk samples rather than emulsions, *i.e.*, there is no surfactant possibly interacting with the glycerol/water solution.

It is important to discuss the different nature of the glycerol–water mixtures that are prepared by isobaric cooling in experiments and MD simulations. The fastest cooling rate employed in the present experiments, *q*_c_ ≈ 100 K s^–1^, is about 7 orders of magnitude slower than the rates employed in the accompanying computational study.^28^ Indeed, the cooling rates employed in the computer simulations are closer to (approximately 2 orders of magnitude faster than) the experimental rates necessary to form hyperquenched glassy water (HGW)^36–38^ and to avoid ice formation. Not surprisingly, ice formation within glycerol–water solutions does not occur upon cooling in MD simulations. Accordingly, the glasses formed upon cooling in the accompanying computational work (at *χ*_g_ < 0.15) are homogeneous vitreous mixtures,^28^ analogous to the glassy mixtures prepared by Suzuki and Mishima.^26^ Instead, the glasses that we form experimentally at these concentrations are phase-separated into ice and glassy mixture domains. Hence, our computational/experimental works cover complementary ranges of cooling rates.

## 3. Results

### 3.1 Pressure-induced formation of HDA in glycerol–water mixtures

**Fig. 1:**
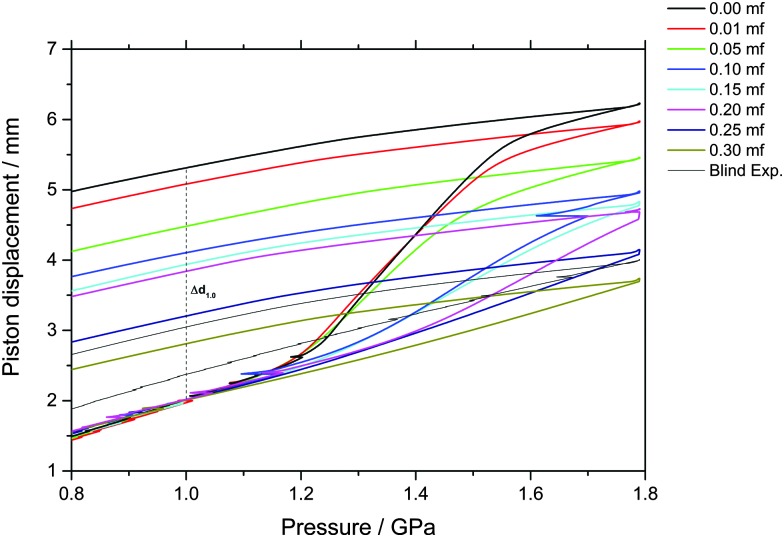
Observation of density changes in crystallized glycerol–water solutions upon compression and decompression at 77 K. Density changes are reported as uniaxial changes in piston displacement for cylindrical samples of diameter 8 mm and initial volume of 600 μl at ambient temperature. The density jump at *P* ≈ 1.2–1.6 GPa and approximately *χ*_g_ ≤ 0.20 is due to PIA of ice in the sample (*i.e.*, ice transforming to HDA). Small kinks during compressions are due to friction, resulting in a clicking noise and some heat release through the sample. Usually this is not relevant, except for the sample at *χ*_g_ = 0.10, in which a few percent of the sample crystallize because of this brief, unwanted heating event. For samples at *χ*_g_ > 0.20, formation of HDA is only visible as slight shifts in curvature.

**Fig. 2:**
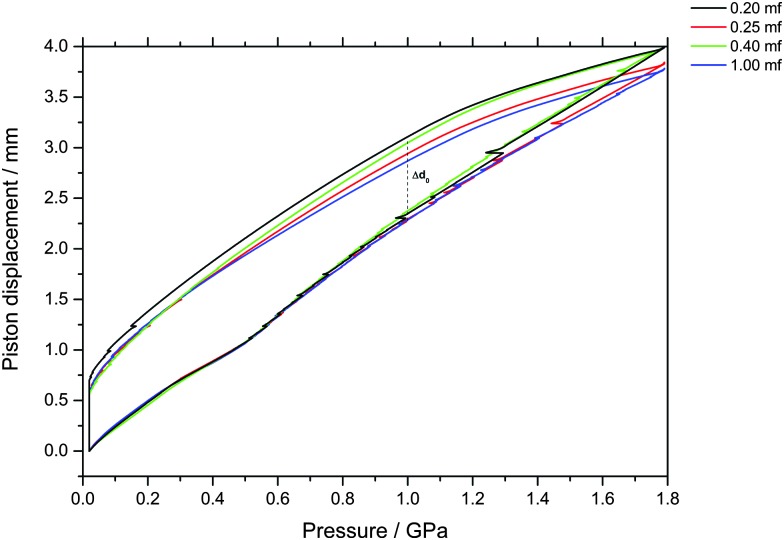
Observation of density changes in vitrified glycerol–water solutions upon compression and decompression at 77 K. Density changes are reported as uniaxial changes in piston displacement for cylindrical samples of diameter 8 mm and initial volume of 600 μl at ambient temperature. Samples of *χ*_g_ < 0.20 could not be vitrified at the fastest cooling rates employed in this study. No signatures of LDA–HDA polyamorphism are found at *χ*_g_ ≥ 0.20.

The piston displacement upon compression of the glycerol–water samples at 77 K is depicted in Fig. 1 and 2. Whereas Fig. 1 shows the changes in volume upon compression of samples that initially contain ice, Fig. 2 shows the changes of volume incurred upon pressurization of vitrified solutions. The range of *χ*_g_ = 0.20–0.25 appears in both figures since the cooling rates employed here (*q*_c_ ≈ 100 K s^–1^–10 K h^–1^) allow for either vitrification or phase-separation (with ice formation) of the sample.

The main point of Fig. 2 is the absence of a sudden density change during the compression of vitrified mixtures at *χ*_g_ ≥ 0.20. Such a sudden density change would signal the existence of a pressure-induced transformation between LDA and HDA, as is the case of pure glassy water. Therefore, our results indicate that there is no glass polymorphism at *χ*_g_ ≥ 0.20, in accordance with the study by Suzuki and Mishima.^26^ They even note the disappearance of polyamorphism at *χ*_g_ ≈ 0.12–0.15. Suzuki and Mishima were able to vitrify more dilute solutions since they were vitrifying emulsified solutions by isobaric cooling at high-pressure (0.3 GPa) conditions, whereas we here vitrified the solutions at ambient pressure. Furthermore, these results are in agreement with the numerical simulation data of the companion paper, which shows that the LDA-like and HDA-like states of the mixtures approach each other as the concentration increases and that, in particular, the distinction between the LDA and HDA states of the glassy solutions disappears at *χ*_g_ > 0.10 (see Fig. 1a and 6a in ref. 28).

By contrast, a step-like change in density is evident upon compression of the phase-separated solution; see Fig. 1. Amorphization of the ice domains within these samples shows up as a relatively sudden densification and results in the sigmoidal curve in Fig. 1. Interestingly, this sudden densification gradually disappears upon increasing glycerol mole fraction. We note that increasing the amount of glycerol also shifts the onset point for amorphization to higher pressure, from about 1.15 GPa in the case of pure water to about 1.30 GPa at *χ*_g_ = 0.20. In addition, the ice-to-HDA transformation broadens with increasing glycerol content, such that the endpoint of the transition can no longer be reached in the range up to 1.8 GPa for mole fractions exceeding 0.20.

**Fig. 3:**
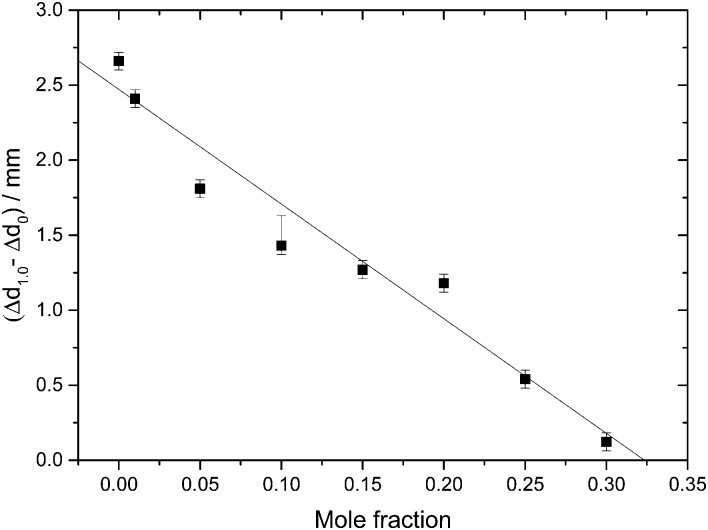
Permanent densification of samples after a compression–decompression cycle calculated as Δ*d*_1.0_ – Δ*d*_0_. Δ*d*_1.0_ (vertical dashed line in Fig. 1), represents the total deformation of the sample plus the steel piston deformation, while Δ*d*_0_ (vertical dashed line in Fig. 2) corresponds to the deformation solely of the steel piston (pertaining to a blind experiment using the 0.40 mf sample, which does not show any permanent densification; see text). The asymmetric error-bar for *χ*_g_ = 0.10 includes the contribution from a small amount of crystalline material, that has formed due to the small pressure-drop, accompanied by shock-wave heating, seen in Fig. 1.

Indirectly, we determine the fraction of ice in the mixtures that transforms to HDA during the PIA experiments (Fig. 1). This is done by reading the difference in the piston position in the up- and down-stroke at 1.0 GPa, Δ*d*_1.0_ (see vertical dashed line in Fig. 1). Δ*d*_1.0_ reflects the sum of the permanent densification of the sample and the densification of the machine itself, Δ*d*_0_, especially due to the steel pistons' deformation. Δ*d*_0_ was determined from a blind-experiment using fully vitrified glycerol solution of *χ*_g_ = 0.40, which is, by contrast to aqueous glycerol of *χ*_g_ ≤ 0.38, not permanently densified in compression/decompression cycles. Δ*d*_0_ was determined to be 0.67 mm from this experiment (see vertical dashed line in Fig. 2). The permanent densification of the sample, *i.e.*, the reduction of the cylinder height Δ*d*_1.0_ minus Δ*d*_0_, is plotted in Fig. 3. In case of pure water (*χ*_g_ = 0), the whole sample transforms to HDA. Evidently, the permanent densification, and thus the fraction of ice experiencing transformation to HDA, decreases with glycerol content and reaches zero at about *χ*_g_ = 0.32. That is, amorphization of ice within the mixtures no longer takes place at *χ*_g_ ≥ 0.32. Rather than that, at *χ*_g_ ≥ 0.32, the sample is elastically compressed and decompressed (residual ice left in the sample, if any, is not able to amorphize).

**Fig. 4:**
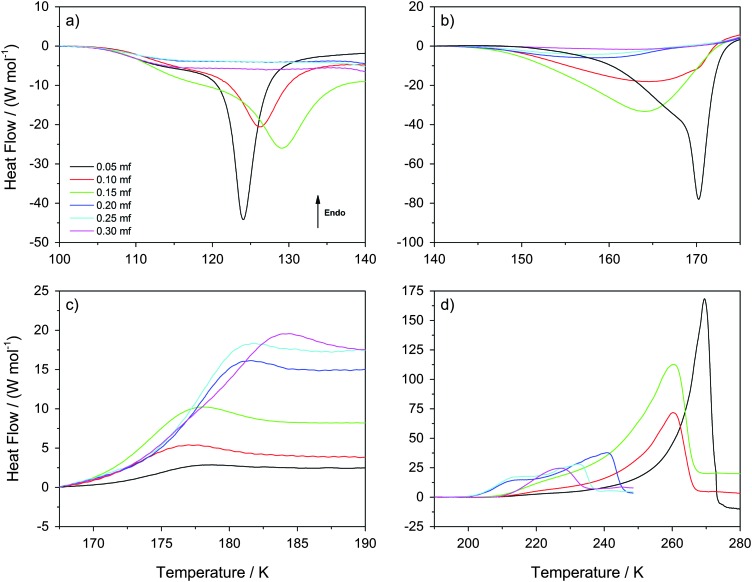
Differential Scanning Calorimetry scans recorded at 30 K min^–1^ heating rate. The four panels (a)–(d) show temperature windows containing (a) HDA → LDA transformations within the sample at *χ*_g_ ≤ 0.15 (but not at *χ*_g_ > 0.15), (b) LDA → ice I_c_ transformations at *χ*_g_ ≤ 0.15 (the system is a mixture of MFCS with HDA at *χ*_g_ > 0.15), (c) the glass transition of MFCS domains, and (d) melting of ice within viscous glycerol–water solution.

The value *χ*_g_ = 0.32 is smaller than the concentration corresponding to the MFCS, *χ*_g_ = 0.38^19,41^ (see also Fig. 7). This is surprising since at *χ*_g_ ≤ 0.38 one would expect that the solution would phase-separate into ice domains and MFCS (*χ*_g_ = 0.38) upon slow cooling. Since our results imply the absence of PIA at 0.32 ≤ *χ*_g_ ≤ 0.38 in the experiments, we conclude that either (i) glycerol–water mixtures do not reach the maximum freeze-concentration in our experiments (with no ice domains in the sample), (ii) the pressure required to trigger PIA exceeds 1.8 GPa, or (iii) some ice precipitates upon cooling from the solution but this ice is distorted by the neighboring glycerol molecules, such that it can no longer be amorphized. We regard (iii) to be the most likely explanation, especially since the maximum freeze-concentration in bulk glycerol solutions was shown to be reached in many experiments in the past, in which the cooling rate was both slower and faster than the slow rate used by us.^17,20,25,33–35^ Also, our *T*_g_ values extracted from Fig. 4c suggest this to be the case (see next section). We call this distorted form of ice the glycerol–water “interphase” (IP), since it should be an ice-like H_2_O layer located at the interface between hexagonal ice domains and glycerol–water domains of *χ*_g_ = 0.38. We prefer the term “interphase” over the term “interface” since we regard the distorted ice to be extended in all three dimensions and not just a thin two-dimensional layer (see also Fig. 6). The term “interphase” emphasizes this 3D nature, whereas “interface” is often associated with a 2D nature. The presence of interfacial ice in cooled glycerol–water samples has been proposed previously (see, *e.g.*, ref. 25). In our experiments, involving compression to *P* = 1.8 GPa at *T* = 77 K, not all of the ice can be amorphized, which supports the theory of this ice being distorted. We do not think that the distorted ice, which is sandwiched between glycerol–water and ice domains, can be amorphized at *P* > 1.8 GPa, since (a) waiting periods at 1.8 GPa do not induce additional amorphization and (b) the onset pressure for amorphization is much lower than 1.8 GPa even for glycerol-rich solutions, *e.g.*, about 1.4 GPa for *χ*_g_ = 0.25. That is, there is indeed a small fraction of the sample that is composed of frozen water, but cannot be amorphized.

That is, compression of samples phase-separated into ice and glassy glycerol–water solution domains at 1 atm, results in a high-pressure material that may contain unchanged glassy glycerol–water solution domains, HDA domains, and/or interphase ice. PIA, and hence HDA-domains, can be observed up to *χ*_g_ = 0.32, where the fraction of HDA-domains decreases linearly with mole fraction of glycerol. In addition, as *χ*_g_ approaches 0.32, both from lower and higher *χ*_g_, the amount of interphase is expected to increase. Between *χ*_g_ = 0.32 and *χ*_g_ = 0.38 (MFCS), PIA is not observed, in spite of ice segregating from the solution. For this reason, we suggest that at these concentrations, any ice domain remains in the sample at high pressure as a high-density, distorted ice-like state (= “interphase”) which resists amorphization even at 1.8 GPa (see also Fig. 6).

### 3.2 DSC analysis of recovered samples at *P* = 1 atm

In this section, we describe the DSC analysis of the samples considered in Fig. 1, which initially contain ice and freeze-concentrated solution domains, *i.e.*, *χ*_g_ < 0.32 (see Fig. 6 for a schematic drawing). The glycerol–water mixtures obtained at *P* = 1.8 GPa are decompressed to ambient pressure under liquid nitrogen in the steel cylinder. They are then transferred (under liquid nitrogen) into a crucible and cold-loaded into the DSC instrument, again, following well established procedures.^6,15^ These samples are then heated at constant pressure at 30 K min^–1^ heating rate. It follows that the samples decompressed back to *P* = 0.1 MPa are composed of glassy glycerol–water solution, domains of HDA, and possibly some “interphase” ice. The calorigrams of these mixtures are shown in Fig. 4a–d for different temperature-intervals.

Fig. 4a shows the temperature region (*T* ≤ 140 K) in which the HDA-to-LDA transformation in pure water is observed. The calorigrams of the samples show two processes that are well-separated, (i) a shoulder with onset temperature of ≈105 K, present at all *χ*_g_, and (ii) a latent heat evolution with onset temperature ≈115 K, present only at *χ*_g_ ≤ 0.15. These two processes were reported for the case of pure water by Handa *et al.* ^4^ According to them, the shoulder in the calorigrams corresponds to a slow enthalpy relaxation process within the strained HDA matrix. In glassy glycerol–water mixtures, the relaxation is observed at a similar temperature, indicating that glycerol–water samples also contain HDA domains that relax to a lower-enthalpy HDA state. The subsequent latent heat evolution characterizes the first-order like nature of the HDA → LDA transition (*χ*_g_ ≤ 0.15). With increasing glycerol content, the latent heat accompanying the HDA → LDA transition decreases, which indicates that the HDA-fraction within the sample decreases. However, the latent heat peak disappears at *χ*_g_ ≥ 0.20, even though HDA domains are present up to *χ*_g_ = 0.32 (see above). Therefore, only at *χ*_g_ < 0.20 the HDA domains within the samples are able to transform to LDA upon heating (*T* < 140 K). Instead, at 0.20 ≤ *χ*_g_ ≤ 0.30, the samples remain as composed of glassy glycerol–water and HDA domains, with some residual interphase ice that increases as *χ*_g_ → 0.30 (*T* < 140 K). We note that the disappearance of the heating-induced HDA → LDA transformation at *χ*_g_ ≥ 0.20 shown in Fig. 4a is fully consistent with the absence of pressure-induced LDA → HDA transformation at *χ*_g_ ≥ 0.20 reported in Fig. 1. More importantly, it is also consistent with the disappearance of the HDA → LDA transition in the experiments by Suzuki and Mishima.^26^ Since Suzuki and Mishima were not studying strained HDA (formed from PIA of ice at 77 K, also called uHDA^42^), but more relaxed HDA (formed *via* the polyamorphic LDA–HDA transition), this finding implies that polyamorphism disappears at *χ*_g_ ≥ 0.20, no matter whether strained or more relaxed HDA-type samples are investigated. Our results are also in excellent agreement with the computer simulations in the companion article.^28^

Interestingly, Fig. 4a shows that the onset temperature for the HDA → LDA transformation shifts to higher temperature with increasing glycerol content. In other words, the HDA-like state is stabilized in the presence of glycerol in the sense that the temperature of its decomposition increases. Combined with the above-mentioned disappearance of polyamorphism at *χ*_g_ > 0.15, this implies the HDA domains within the samples become thermodynamically more stable than the LDA domains at *χ*_g_ ≥ 0.20. In other words, by contrast to the pure water case, HDA is thermodynamically more stable than LDA at *χ*_g_ ≥ 0.20 even at ambient pressure. That is, polyamorphism disappears at positive pressure at *χ*_g_ ≥ 0.20.

**Fig. 5:**
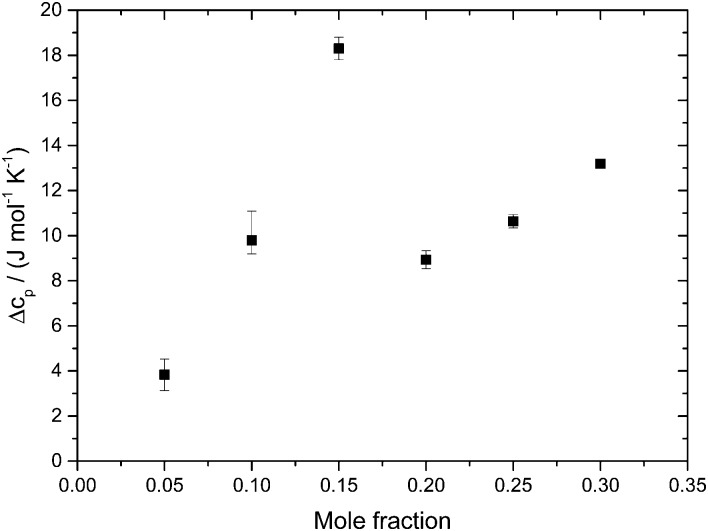
Difference in heat capacity, Δ*c*_P_(*χ*_g_), at 100 K (in the HDA-like state) and 140 K (in the “LDA”-like state). Δ*c*_P_(*χ*_g_) shows two linear regimes; the jump-like change near *χ*_g_ ∼ 0.15–0.20 indicates disappearance of the HDA-to-LDA transformation (only found *χ*_g_ ≤ 0.15).

Fig. 5 shows the evolution of the heat capacity difference, Δ*c*_P_, at about 100 K and 140 K, *i.e.*, the difference before and after the HDA → LDA transition exotherm observed at *χ*_g_ ≤ 0.15. Δ*c*_P_(*χ*_g_) exhibits two separated rather linear regimes, at *χ*_g_ ≤ 0.15 and *χ*_g_ ≥ 0.20, corresponding to the presence/absence of heating-induced HDA → LDA transformations. Δ*c*_P_ increases at *χ*_g_ ≤ 0.15, in agreement with the thermodynamics of binary mixtures in the two-phase (HDA/LDA) domain.^43^ At *χ*_g_ ≈ 0.15 there is a sudden jump in Δ*c*_P_, indicating the sudden disappearance of polyamorphism and the transition to the one-phase domain. This is indicated in Fig. 7, which shows the phase diagram of glycerol–water. At 1 atm there is a two-phase domain, between about 125 K and 140 K (see Fig. 4a), extending up to *χ*_g_ ≈ 0.15. At *χ*_g_ > 0.15 the HDA–LDA transition disappears, and so only a single glassy phase, which resembles HDA is observed. For solutions in the range 0.20 ≤ *χ*_g_ ≤ 0.30, only the extended relaxation process contributes to the heat capacity change increase (see Fig. 4a).^8^ A similar cusp-like change of heat capacity from high values, at low mole fraction, to low values, at high mole fraction, is predicted by Biddle *et al.* (see Fig. 6 in ref. 43), and was previously observed in NaCl–water solutions by Archer and Carter.^44^ They interpret such a behavior to be caused by the suppression of polyamorphism and water anomalies at sufficiently high mixing ratios.

**Fig. 6:**
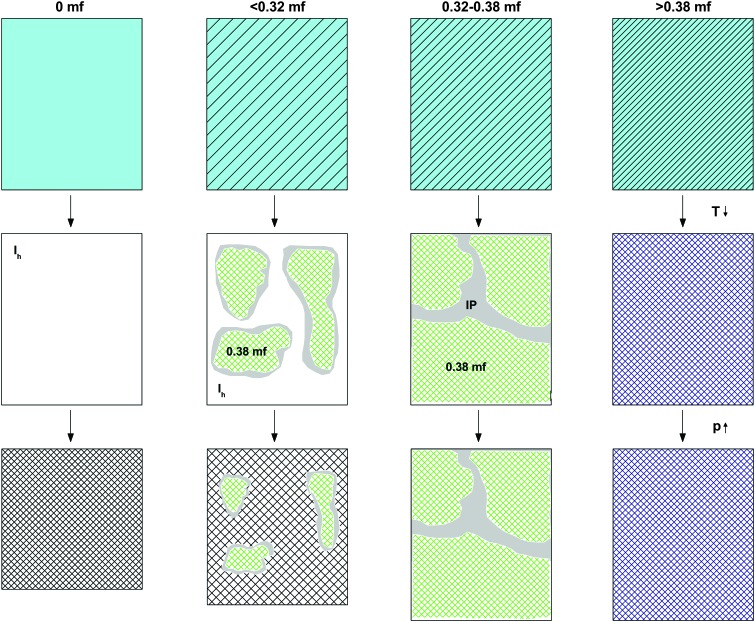
Cartoon indicating the density of the initial solution at room temperature and normal pressure (first line), the phase separation and formation of interphase upon cooling to 77 K at ambient pressure (second line), and the pressure-induced amorphization at *T* = 77 K (third line) for different *χ*_g_ ranges. White, light blue, and grey colors represent, respectively, ice, homogeneous glycerol–water solution, and ice “interphase” (IP). Hatching indicates increase in glycerol concentration. Green represents glassy domains of MFCS (*χ*_g_ = 0.38).

**Fig. 7:**
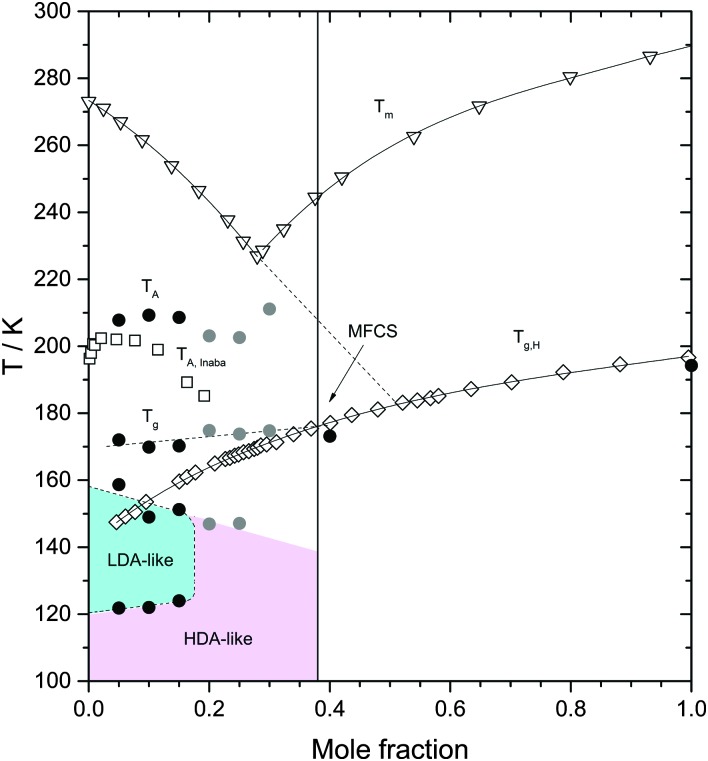
Heating induced transformations in glycerol–water solutions at 1 atm. Triangles are the (equilibrium) melting temperature *T*_m_ from ref. 39; diamonds are the glass transition temperature of the homogeneous, fully vitrified solutions *T*_g,H_, at *χ*_g_ ≥ 0.38, and the glass transition temperature of the maximally freeze-concentrated solution *T*_g MFCS_, at *χ*_g_ < 0.38, from ref. 40. Squares are the melting temperature of ice domains within glycerol–water mixtures, *T*_A_, from ref. 19. Filled symbols are extracted from Fig. 4 in this work. Black and grey circles were obtained from fast and slowly cooled solutions, respectively. The concentration corresponding to the MFCS is marked by a vertical line and an arrow. Glass polymorphism occurs only at *χ*_g_ ≤ 0.15.

Fig. 4b shows the calorigrams in the temperature range 140–175 K in which (pure) LDA crystallizes. At these temperatures, we find that samples containing LDA domains (*χ*_g_ ≤ 0.15) exhibit a strongly exothermic crystallization, while samples at *χ*_g_ ≥ 0.20 show only a weak and very broad exotherm. In the case of pure water, LDA crystallizes to cubic ice releasing a latent heat of approximately Δ*H* = –1.30 kJ mol^–1^.^4,45–47^ Fig. 4b shows that, adding glycerol reduces the latent heat associated to the LDA → ice transformation. On the basis of per mol of solution (not per mol of water), the latent heat released decreases from Δ*H* = –0.91 kJ mol^–1^ at *χ*_g_ = 0.05 to Δ*H* = –0.83 kJ mol^–1^ at *χ*_g_ = 0.15, and it is very small at *χ*_g_ ≥ 0.20 (–0.2 < Δ*H* < –0.1 kJ mol^–1^). This decrease is not only due to the decrease of water content; the fact that Δ*H* → 0 at *χ*_g_ ≥ 0.20 is mainly due to the inability of HDA to transform to LDA at *T* < 140 K (*i.e.*, there is no LDA in the samples at these concentrations). It also follows from Fig. 4b that any interphase ice in the samples is not able to crystallize either and hence, interphase ice remains within the sample even at high temperatures. Regarding the residual HDA at *χ*_g_ ≥ 0.20, we see a very broad and weak peak in Fig. 4b, which suggests that it transforms slowly to ice at 140–170 K. We think this is the case because HDA gradually becomes less viscous, and the crystallization dynamics becomes fast enough to enable segregation. In fact, the *T*_g_ for HDA at 1 atm is 116 K, and so “HDA”-domains are in the less and less ultraviscous liquid state upon heating to 140–170 K called high-density liquid (HDL). Therefore, the samples at *T* ≈ 170 K (Fig. 4b) consist of domains of MFCS (*χ*_g_ ≈ 0.38) combined with ice domains and “interphase” ice, regardless of glycerol concentration. The amount of HDA/HDL (at *T* < 170 K) or ice (at *T* > 170 K) approaches zero as *χ*_g_ → 0.32, and the amount of interphase ice increases.

Interestingly, adding glycerol to the mixtures also reduces the LDA → ice onset temperature, *i.e.*, the exothermic peak in Fig. 4b shifts to lower temperatures with increasing *χ*_g_. One way of explaining this finding is that the pure LDA domains in glycerol–water solutions experience a glass transition (hidden in the baseline-noise). We surmise this glass transition is shifted to lower temperature because of the contact with glycerol–water domains, taking place below 136 K, which is the *T*_g_ for pure LDA.^47^ Once the ultraviscous liquid state (low-density liquid, LDL) is reached in these domains, crystallization dynamics speeds up, shifting the LDA → ice transition temperature to lower temperatures. This indicates that glycerol is miscible not only with HDA, but also with LDA. The broadening of the peaks with increasing glycerol content indicates that the viscosity is higher in the presence of glycerol, and so the LDA → ice transition is more and more kinetically controlled. This is in contrast to the situation in LiCl solutions, in which the ions are only soluble in the HDA-matrix but not in the LDA-matrix.^48–50^ In case of glycerol, hydrogen bonds can form between glycerol and water both in the HDA-like and in the LDA-like state. At *χ*_g_ ≥ 0.20 the HDA-like hydration of glycerol only needs to expel a small amount of water to reach the maximal freeze concentration, which is unable to crystallize further.

Fig. 4c shows the calorigrams in the temperature range 167–190 K. At these temperatures, we observe that all samples experience a glass transition at *T*_g_ ≈ 172 K, close to the glass transition temperature of the MFCS (*T*_g_ ≈ 175 K for rates of 20 K min^–1^ ^40^). Hence, this glass transition is due to the glass-to-liquid transformation of the glassy glycerol–water domains. The fact that such a glass transition occurs at almost the same temperature, independent of *χ*_g_, shows that the composition of the glycerol–water patches is identical, namely the MFCS (see Fig. 7). This is in good agreement with the data from Harran^40^ who used a rate of 20 K min^–1^ in his work. However, the fraction of MFCS patches increases with increasing *χ*_g_ and so the change in heat capacity, Δ*c*_p_, at 172 K also increases with *χ*_g_. In other words, the Δ*c*_p_ observed at the glass-transition can be used to calibrate the mass of glycerol and water. Similar findings were reported by Popov *et al.* for solutions ranging for 0.025 < *χ*_g_ < 0.15 (see their Fig. 5a, inset),^25^ even though they did not use high-pressure equipment in their work. The endpoint of the glass transition range is located at *T* = 180–185 K. Above that, the concentrated patches of glycerol–water are in the viscous liquid state, whereas the water domains are still in the solid, icy state.

Fig. 4d shows the calorigrams at high temperatures at which the ice domains melt. The melting of this ice corresponds to the peak observed in Fig. 4d. These peaks are accompanied by a characteristic low-temperature tail of debated nature, including a shoulder on top of the tail near ≈210 K, which is sometimes called “drift anomaly”. The onset temperature of the drift anomaly is labeled *T*_A_ in Fig. 7. Results from our work are compared with results obtained earlier by Inaba and Andersson.^19^ As also discussed by them,^19^ the tail itself can be explained in terms of the melting of ice crystals within the viscous glycerol solution. Upon melting, the solution becomes gradually diluted and hence, the melting temperature shifts as the melting proceeds. Ultimately, the highest melting temperature reached corresponds to the melting temperature of the initial, overall composition of the solution. While the origin of the melting tail itself is generally accepted, explanations of the drift anomaly are disparate. One explanation involves two freeze-concentrated solutions (FCS_1_ and FCS_2_) of different concentration, as *e.g.*, also advanced in case of aqueous citric acid solutions.^51,52^ In this view, the shoulder represents the glass transition due to devitrification of FCS_2_. Another interpretation was put forward by Inaba and Andersson based on ice crystallization upon warming followed by ice dissolution.^19^ Also a two-dimensionally ordered domain of ice could be at the origin of this feature in the calorigram.^20^

We interpret the drift anomaly in glycerol–water to indicate melting of the “interphase” ice. The interphase melts at lower temperatures than bulk ice because of its distorted, high-enthalpy nature. Inspecting the change of *T*_A_ with *χ*_g_, one can note that *T*_A_ first increases and then it decreases up to *χ*_g_ ≈ 0.25. This effect can be explained by the entropy difference between distorted ice and glycerol–water, which decreases with increasing glycerol content. At *χ*_g_ ≈ 0.25 there is a minimum (see Fig. 7), *i.e.*, at *χ*_g_ > 0.25, *T*_A_ increases with increase in glycerol content. We suggest, that this turnaround is due to the different nature of the “interphase”: At *χ*_g_ > 0.25 the “interphase” is sandwiched between glycerol–water domains from both sides, whereas it is in contact with glycerol–water only from one side at *χ*_g_ < 0.25. As a consequence the interphase reaches maximum distortion and higher temperatures are now required to compensate the enthalpic destabilization effect.

## 4. Discussion and conclusion

Our experiments indicate that pressure-induced glass polymorphism does not exist in glycerol–water at *χ*_g_ ≥ 0.20 when vitrified at 1 atm, *i.e.*, there is no LDA → HDA-type transition. This is in agreement with the work of Suzuki and Mishima,^26^ who vitrified emulsified solutions under pressure (0.3 GPa) and found no glass polymorphism at *χ*_g_ ≥ 0.10–0.12 *i.e.*, they found no HDA → LDA-type transition. In both experiments, this is because the LDA-like state can no longer be accessed at high *χ*_g_. Our results are also in agreement with MD simulation of the companion paper that show no glass polymorphism in hyperquenched solutions for approximately *χ*_g_ ≥ 0.10.^28^

Most of our experiments involve the study of PIA and heating-induced transformations of low-concentrated solutions. In order to explain our observations, we discuss separately the transformations observed (i) during cooling and compression of the solutions (see Fig. 6), and (ii) upon heating the samples decompressed at 1 atm (see Fig. 7). We suggest the phenomenology sketched in Fig. 6 to take place upon cooling and pressurizing the glycerol–water solutions. In the absence of glycerol, we find the phenomenology established by Mishima *et al.* about 30 years ago.^1,2^ That is, liquid water (light blue in Fig. 6) crystallizes to small crystallites of hexagonal ice (white in Fig. 6) upon cooling, which experience PIA to form HDA (hatched-white in Fig. 6) at 77 K. In the presence of glycerol, the density of the liquid phase increases (light-blue-hatched in Fig. 6). Upon cooling at concentrations *χ*_g_ ≤ 0.38, the maximally freeze-concentrated solution (*χ*_g_ = 0.38; green hatched in Fig. 6) forms as domains coexisting with hexagonal ice domains. As expected, the size of the glycerol–water domains (ice) increase (decrease) with increasing *χ*_g_. At 77 K glycerol–water domains are in the glassy state. The interface between the crystalline ice and the glassy glycerol–water domains is sketched in grey and corresponds to the “interphase” ice. This interphase can be imagined as ice crystallites showing a distorted structure because of the interaction with the glassy, glycerol-rich patches. Upon pressurization, only the bulk-like ice domains may be affected and experience PIA to form HDA at *χ*_g_ ≤ 0.32, while the distorted ice remains in its strained, high-density state (which is maybe close to the HDA density even before compression). Also the glassy glycerol–water domains are unaffected by the pressure treatment.

The observation that HDA no longer forms during compression at *χ*_g_ > 0.32 is striking (see Fig. 3). Naively, one would expect HDA to form as long as there are icy domains left, which should occur up to the MFCS concentration, *χ*_g_ = 0.38. We interpret the absence of HDA at 0.32 < *χ*_g_ ≤ 0.38 to indicate the formation of the above-mentioned interphase of highly distorted ice in contact with the MFCS. At *χ*_g_ = 0.38 the distorted ice occupies about 1/6 of the whole volume, *i.e.*, it has to be a spatially extended, 3D structure, and cannot just be a 2D layer. This is indicated in Fig. 6, third column, by the absence of ice (white domains). Instead, all the MFCS patches (green) are linked with one another by this distorted ice phase (grey “interphase”, labeled IP). An alternative explanation of our observations would be that the patches do not reach the MFCS, but are freeze-concentrated to less than a mole fraction of 0.38 because of viscosity and diffusion limitation. However, this is hard to reconcile with the observation that the glass transition of the glycerol–water patches is observed at the same temperature (*T*_g_ ≈ 172 K in Fig. 4b) regardless of the mole fraction of the initial solution. Upon pressurization to 1.8 GPa at 77 K, these linked patches remain unaffected. At mole fraction of *χ*_g_ ≥ 0.38 the entire sample vitrifies, without ice formation and without interphase (fourth column in Fig. 6). In addition, these samples are unaffected by pressure up to 1.8 GPa.

The effects of heating the recovered samples at *P* = 1 atm are summarized in Fig. 7. For comparison, Fig. 7 includes the melting temperature *T*_m_(*χ*_g_) of glycerol–water mixtures^39^ and the glass transition of homogeneous glassy mixtures, *T*_g,H_(*χ*_g_).^40^ Our values of *T*_g,H_(*χ*_g_) for the homogeneous mixtures at *χ*_g_ = 0.4 and 1.0 are in very good agreement with ref. 40 (Fig. 7). They are lower by 2–3 K because of the slower rate employed in the present study. Upon heating the samples at *χ*_g_ ≤ 0.15, the HDA domains transform to LDA domains at *T* = 120–125 K, which, upon further heating, transform to ice at *T* = 150–160 K. At even higher temperatures, the MFCS domains of the samples devitrify; the corresponding glass transition temperature of these domains is *T*_g_ ≈ 172 K, consistent with the value of *T*_g,H_ for *χ*_g_ = 0.38 (see Fig. 7) and hence, reinforcing the view that the glycerol–water domains in the samples are all MFCS, independently of the initial concentration of the solutions. Upon further heating, ice domains trapped in ultraviscous glycerol–water solution starts to melt slowly near 190 K (not indicated in Fig. 7, visible in Fig. 4b as slight deviation from baseline). The “drift anomaly”, *i.e.*, an additional signal superimposed on the melting tail, occurs at *T*_A_ ≈ 200–210 K. *T*_A_ decreases with *χ*_g_ at *χ*_g_ ≤ 0.15.

Upon heating the samples at *χ*_g_ ≥ 0.20, we do not find polyamorphism, *i.e.*, HDA domains do not transform to LDA. Instead, they remain as HDA domains within the sample up to *T* ≈ 150 K. It follows that the region of LDA-existence summarized in Fig. 7 is at odds with the possibility of a liquid–liquid transition at *χ*_g_ = 0.17, as reported by Murata and Tanaka.^24^ At 150 K the domains are still of HDA-structure. In terms of dynamics “HDA” domains are well above *T*_g_(HDA) = 116 K, *i.e.*, in the ultraviscous HDL state, which slowly transforms into ice upon warming. Crystallization rates for (pure, solute-free) HDA/HDL were reported in our recent work as a function of pressure.^53^ Assuming the electrostrictive force exerted by the solute molecules is similar to the effect of pressure these data suggests crystallization on the time scale of minutes/hours at 150 K, compatible with the DSC observations made here on glycerol–water. As for *χ*_g_ ≤ 0.15, we find that further heating at *χ*_g_ ≥ 0.20 induces the glass transition of the glassy–water domains, MFCS, within the sample, again, at *T* ≈ 172 K. This is followed by melting of the ice/interfacial ice above about *T* = 190 K, as is the case at *χ*_g_ ≤ 0.15. Interestingly, *T*_A_ starts to break the trend of its decrease and starts to increase with *χ*_g_ at *χ*_g_ > 0.25, which is the location of the *T*_A_ minimum. We interpret this minimum to arise because the distortion of the “interphase” ice is largest, when the interphase is in contact with glycerol–water solution from all sides. At decreasing *χ*_g_ the “interphase” is less and less in contact with glycerol–water, but more and more in contact with ice, making the “interphase” less and less distorted. We are unable to distinguish whether “interphase” ice is more ice-like and actually melts (which would imply the additional signal in Fig. 4d to be an endotherm) or whether the interface is more glass-like (which would imply the additional signal to be a second glass transition). Inaba and Andersson have interpreted this anomaly to be due to cold crystallization followed by ice dissolution.^19^ In any case, at a mole fraction of 0.32, the distorted, nanocrystalline or glassy interphase is large enough so that no undistorted ice is left.

This interpretation of ice melting within the samples is consistent with the calorimetry work by Andersson *et al.*, who also see the need of an interface to understand their data.^20^ They call this interface to be a “two-dimensionally ordered structure of ice”. From our experiments the interface seems to occupy up to 1/6 of the volume, and so we can hardly think of it as a two-dimensional interface. Our interpretation is largely consistent with the one presented by Popov *et al.* as a function of glycerol content. However, in our case we do not need to treat separately the range between *χ*_g_ = 0.00 and 0.30 (0.28) into two parts, as they do. This separation is only necessary since, at a fixed cooling rate, more dilute solutions crystallize, whereas more concentrated solutions vitrify. In our case, we set the cooling rate slow enough to avoid vitrification also for the more concentrated solutions up to *χ*_g_ = 0.30.

We conclude by noticing that the cooling rates explored in this work are slower than those accessible in computer simulations, including the MD simulations of the companion paper.^28^ For a closer comparison between the experiments and MD simulations, it is necessary to either (i) crystallize solutions at 0.00–0.30 at slow rates using MD simulations, or (ii) to experimentally, vitrify the solutions at ultrafast rates using our hyperquenching technique. At present, only option (ii) is feasible and this will be the subject of a future study.
